# What Is A Good Editorial?

**DOI:** 10.4103/0973-1229.27600

**Published:** 2006

**Authors:** Ajai Singh, Shakuntala Singh

What are the qualities that distinguish a good editorial? Are there certain essential attributes? What should a good editorial do to a reader, and what not?

These are some crucial questions that every editor, editorial board member, journal and its policy makers should decide for themselves and their respective publications. To that extent it is individual, and some may consider it the internal matter of the publication. However, a broad consensus on certain essential parameters maybe desirable, even essential, if the individual has also to be a significant part of the wider knowledge corpus which all editorials pooled together represent.

We wonder if ever an exercise to publish all editorials of a certain publication has been undertaken, say over a five or ten year period. Or for that matter, say hundred editorials from hundred different editors. It may make for fascinating reading. We hope some smart publisher is reading this. It is possible editorials of one editor may have been compiled and published in book form. That itself is not uninteresting. But the flavour of different edits by different authors is, well, in a class by itself. Wonder if it has been attempted ever?

Of course we know why it may not have been done. Editors, by and large, are reticent people, with a magnified sense of their own importance. Well, this may hurt some, but before they jump at our throats, let us clarify that we belong there as well (The group of editors, reticent, and pompous.). Hence, they may be willing to publish a book of their own edits, but maybe averse to a book with multiple editors as co-authors. Maybe some smart publisher should manage it. He will make his bucks, for sure. And the readers, including fellow editors, will hugely enjoy the fare offered, as they savour the stuff that goes into edit writing. And a second important service will be to help deflate some editorial egos, much in need of puncturing, as so many readers would vouch for.

Enough of that for the present, for we must concentrate on the questions raised at the beginning of this essay. And we hope fellow editors can take some ego puncturing sportingly. Are they not doing it to their writers all the time? It helps to get to the other side of the fence on occasions. Never mind, for those who feel sour faced, there is solace. Their position in the periodical will ensure their ego builds up with some speed once again.

## Opinion Maker, Reconciliatory, Balanced and Crusading

The very first criterion is that a good editorial is an opinion maker. If it is based on evidence, so much the better. But it analyses evidence rather than produces it. Of course what it analyses can be the basis of the production of new evidence. But it is more like the ‘Results and Discussion’ that follow ‘Materials and Method’ in a research paper in so far as it is an objective analysis. However, it goes beyond an analysis. It must necessarily also express an opinion. It must attempt to critically analyse and sift from the various opinions, analyses and evidences floating around. It must present a refreshing perspective on an issue so as to retain balance when writings get opinionated; and/or stir up the crotchety and crusty when scientific/creative stupor sets in. Moreover, a good editorial is contemporary without being populist. It tackles recent events and issues, and attempts to formulate viewpoints based on an objective analysis of happenings and conflicting/contrary opinions.

An editorial is predominantly about balance. But that does not prevent it from occasionally stirring things up, when such is the need. Hence a hard-hitting editorial is as legitimate as a balanced equipoise that reconciles apparently conflicting positions and controversial posturings, whether amongst politicians (in news papers), or amongst researchers (in academic journals).

All said and done, the element of balance can never be lost. For that, it certainly helps if an editor is a balanced individual by temperament as well. However, let it not mean that balance in temperament excludes crusading zeal. Most editors of some merit have the latter in reasonable quantity, although they may play it down, or publicly make a mockery of it, since it is the in thing to do (the mockery, not the crusading). Moreover, denial can be a strong defense mechanism, as much in editors as in the rest of humanity.

Make no mistake about it. Forget the loud protestation to the contrary. Scratch the surface of any good editor who enjoys his job, and a crusader will shine through.

To sum up, a good editorial is either one or more of the following: it is an opinion maker, it is reconciliatory between contrary viewpoints or standpoints, it is balanced in its analysis of evidence and events, and it is, manifest or otherwise, crusading in its thrust.

## The Language

An editorial is traditionally written in a literary style. While it is difficult to define what a literary style is, let us say it is one in which thought is well clothed in language. So well that an editorial may make for a literary piece in literature, aside and apart from its factual or scientific content. However, having said that, it must be noted that an editorial is not only a literary piece. It must also express a firm and balanced opinion on something, an opinion that clarifies the muddle into which committed writers and researchers may lead the reader. At no stage must the language overshadow the thought, however. That is a subtle distinction to maintain. The thought may be embellished by language, not drowned in it. It is very much like a beautiful lady in an equally beautiful dress. Her beauty must be accentuated by the dress. She should not get drowned, or over shadowed, by it, for then the whole exercise is counterproductive. Like when a model becomes just a peg to drape a dress on. That is a distinct danger a good editorial writer must beware of. But, even if it be so, we may note that an editor with a literary flair can make even a humdrum issue vibrate with his unique touch.

In sum, then, language is an important accessory, but never the main thing.

## The After Taste

Like the dessert after a good meal leaves an, in fact decides the, after taste, a good editorial must also be careful to leave a good after taste. This is one in which the reader is held to the piece and retains his interest right till the end. So the piece has to be sufficiently brief to hold his attention, and equally entertaining to hold his attention so that the wholesome is imbibed. It must be such that the reader feels enlightened, or empowered, or helped in forming his own opinion on an issue. While a good editorial expresses an opinion, it does not force it down the throat of the reader. It is subtle enough to appeal to the good sense of the knowledgeable reader without forcing him to toe its line. This is its real test.

The feeling after a good editorial is done with is one of profundity. It is of being in the presence of an enlightened being. It is of feeling ennobled and charged to do something worthwhile, or feel reconciled from a knotty or vexing thought process. It must, moreover, want you to give it a second read. Like wanting a second helping of a good dessert. And want to read further editorials by the same author. Like wanting ones favourite dessert after a meal.

## Summing Up

A good editorial should express an opinion without being opinionated. It should teach without being pedagogic. It should transform without being evangelical. It should engulf without drowning. It should motivate to action without making you dictatorial. It should enlighten without getting you dogmatic, prejudiced and egotistical.

The last, and probably most important, a good editorial should be brief.

An article about a good editorial should also, if possible, be brief.

We hope this was.

